# Identification and In Silico Prediction of Anticoagulant Peptides from the Enzymatic Hydrolysates of *Mytilus edulis* Proteins

**DOI:** 10.3390/ijms19072100

**Published:** 2018-07-19

**Authors:** Meiling Qiao, Maolin Tu, Hui Chen, Fengjiao Mao, Cuiping Yu, Ming Du

**Affiliations:** 1 National Engineering Research Center of Seafood, School of Food Science and Technology, Dalian Polytechnic University, Dalian 116034, China; qiaomeiling513@163.com (M.Q.); realcrital@126.com (H.C.); fengjiaomao@163.com (F.M.); 18340854513@163.com (C.Y.); 2 Department of Food Science and Engineering, Harbin Institute of Technology, Harbin 150090, China; tumaolin012@163.com

**Keywords:** *Mytilus edulis*, enzymatic hydrolysis, UPLC-Q-TOF-MS/MS, molecular docking, anticoagulant activity

## Abstract

*Mytilus edulis* is a typical marine bivalve mollusk. Many kinds of bioactive components with nutritional and pharmaceutical activities in *Mytilus edulis* were reported. In this study, eight different parts of *Mytilus edulis* tissues, i.e., the foot, byssus, pedal retractor muscle, mantle, gill, adductor muscle, viscera, and other parts, were separated and the proteins from these tissues were prepared. A total of 277 unique peptides from the hydrolysates of different proteins were identified by UPLC-Q-TOF-MS/MS, and the molecular weight distribution of the peptides in different tissues was investigated by sodium dodecyl sulfate-polyacrylamide gel electrophoresis (SDS-PAGE). The bioactivity of the peptides was predicted through the Peptide Ranker database and molecular docking. Moreover, the peptides from the adductor muscle were chosen to do the active validation of anticoagulant activity. The active mechanism of three peptides from the adductor muscle, VQQELEDAEERADSAEGSLQK, RMEADIAAMQSDLDDALNGQR, and AAFLLGVNSNDLLK, were analyzed by Discovery Studio 2017, which also explained the anticoagulant activity of the hydrolysates of proteins from adductor muscle. This study optimized a screening and identification method of bioactive peptides from enzymatic hydrolysates of different tissues in *Mytilus edulis*.

## 1. Introduction

Bioactive peptides, as functional ingredients and pharmaceutical agents, have attracted much more attention in recent years [1,2,3]. With the development of new isolation and identification technologies, the study of the relationship of sequence and structure of bioactive peptides has rapidly evolved [3,4].

*Mytilus edulis* is a typical marine bivalve mollusk. Many kinds of nutritional and pharmaceutical benefits of *Mytilus edulis* have been reported, such as nourishing the liver and kidneys, adjusting blood pressure, curing night sweats, dizziness and impotence, and so on [5]. More and more bioactive peptides from the *M. edulis* whole body rather than different tissues, such as antimicrobial peptides [6,7], anti-inflammatory peptides [5], antioxidant peptides [8,9] and anticoagulant peptides, have been reported in recent years. Meanwhile, the bioactive peptides from different tissues of *M. edulis* are identifiable [10].

Thrombosis may cause serious complications such as an increase in portal venous pressure and an intestinal infarction. Previous surveys have indicated that the majority of serious diseases are thrombotic diseases, which may result in sudden death or long-term disability [11]. In recent years, bioactive peptides have received increasing attention. Similarly, there is an increasing need for anticoagulant and antithrombotic peptides to cure thrombosis. In these booming fields, those natural peptides with thrombin-inhibitory activity have attracted much more attention and have been evaluated extensively. Therefore, it is necessary to elaborate the preparation methods of target peptides. Indeed, blood clotting is a complicated physiological process controlled by a series of proteolytic reactions with comprehensive interactions [12,13]. The blood coagulation pathway involves the interaction of many plasma serine proteases known as blood clotting factors [5]. Therefore, the molecular mechanism of protein/peptide recognition has very important implications in the fields of biology, medicine, and pharmaceutical sciences [14]. Molecular docking technology has been widely used in this field in recent years [15,16,17].

The antithrombotic activity of *M. edulis* hydrolysate has been reported in several studies [6]. However, anticoagulant peptides were rarely isolated from *M. edulis*, and interactions between the peptide and thrombin have not been reported. The aim of this work was to prepare bioactive peptides from different tissues of *M. edulis* by trypsin digestion, and several possible anticoagulant peptides were evaluated by molecular docking and Peptide Ranker.

## 2. Results and Discussion

### 2.1. Distribution of Proteins from Different Tissues

**Figure 1:**
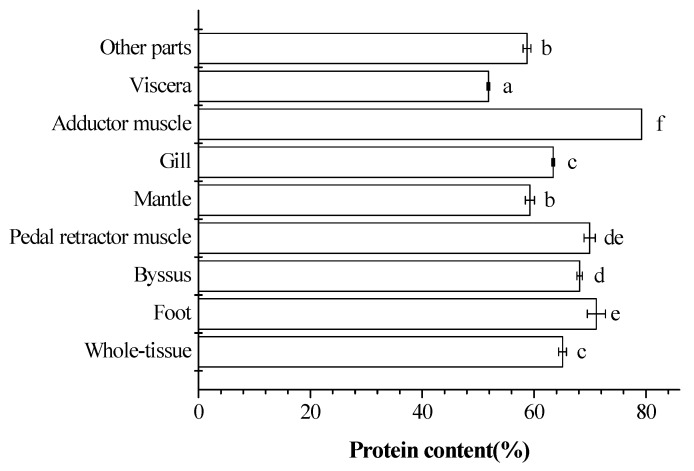
Eight different parts of *Mytilus edulis* tissues, i.e., foot, byssus, pedal retractor muscle, mantle, gill, adductor muscle, viscera, and other parts, from *Mytilus edulis* were separated and prepared. The protein content of different tissues was measured by the Kjeldahl method. Values are mean ± SD (*n* = 3–6). Different letters beside the bars represent a significant difference between the values.

The protein content of the different samples was shown in Figure 1. The adductor muscle, foot, pedal retractor muscle, byssus, gill, mantle, other parts, and viscera were sorted depending on the protein content. The protein content of the powder of S0 was 68%. S1, S2, S3, S4, S6, S7, and S8 had significant differences compared with S0. S6 was significantly higher than S0. S2, S4, and S8 were significantly lower than S0. When the total tissue was considered to be 100%, the dry weight of the different tissues will be 65.08 ± 0.71, 71.13 ± 1.62, 68.14 ± 0.48, 69.92 ± 1, 59.25 ± 0.83, 63.41 ± 0.23, 79.22 ± 0, 51.84 ± 0.25, 58.73 ± 0.72 percent, respectively.

**Figure 2:**
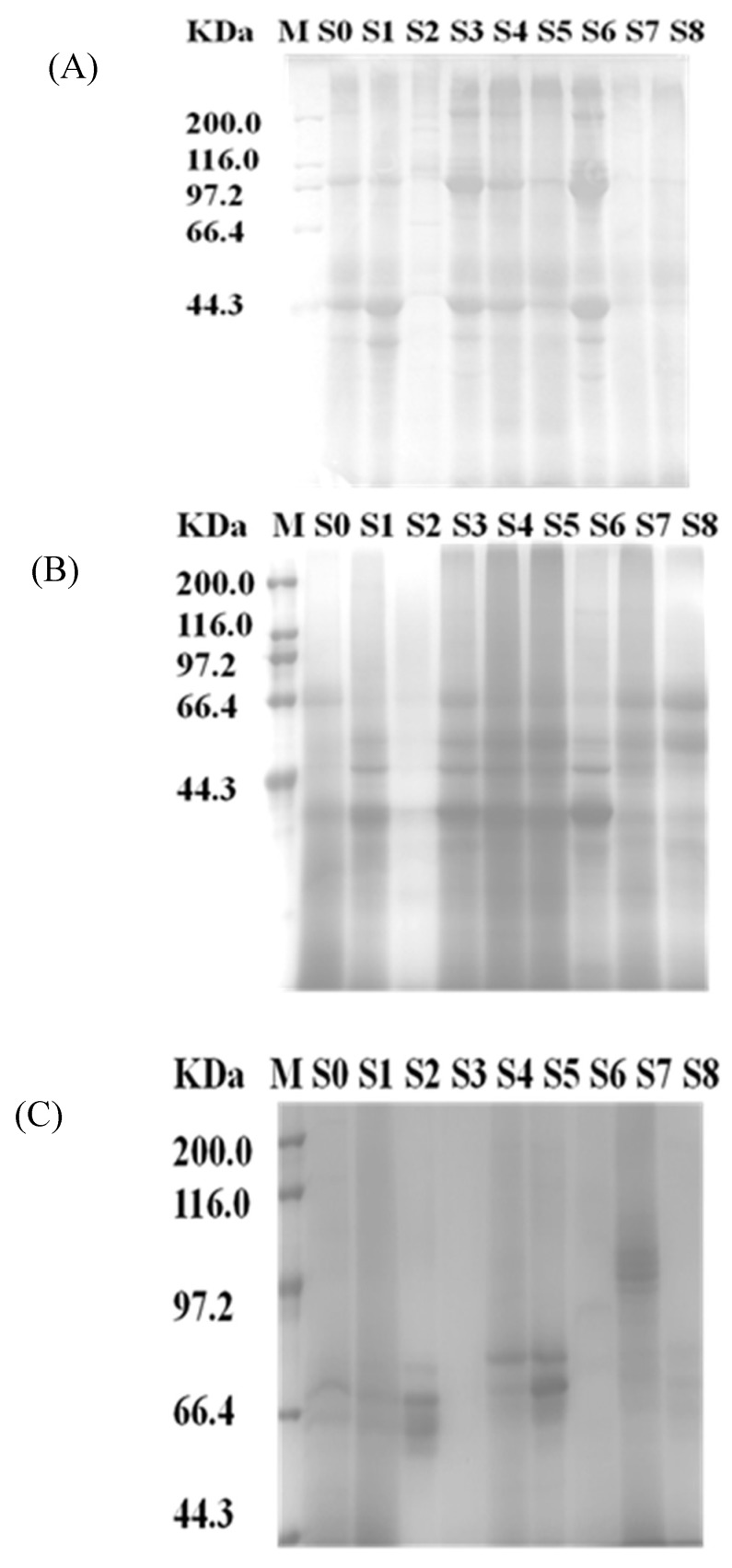
(**A**) Soluble proteins in 45 °C water at pH 8.5; (**B**) soluble proteins in 25 °C water at pH 7.0 (**C**) proteins and peptides in the enzymatic hydrolysates. S0—Whole tissue; S1—Foot; S2—Byssus; S3—Pedal retractor muscle; S4—Mantle; S5—Gill; S6—Adductor muscle; S7—Viscera; S8—Other parts.

As shown in Figure 2, the proteins of byssus, viscera, and others about 40 kDa proteins were more noticeable than other samples. Dissolved protein concentrations were 7.45 ± 0.11, 4.53 ± 0.21, 2.88 ± 0.28, 5.71 ± 0.05, 5.47 ± 0.08, 5.83 ± 0.09, 5.57 ± 0.07, 4.05 ± 0.18 and 5.57 ± 0.06 mg/mL, respectively, as determined by the BCA method (*R*^2^ ≥ 0.998). It was noteworthy that the protein concentration of byssus was too low to affect later enzymatic hydrolysis. As shown in Figure 2A,B, there was different protein distribution, i.e., the protein band of 100 kDa in 25 °C water (pH 7.0) was not soluble in 45 °C water (pH 8.5), which probably contributed to the different peptide identification. The effect of enzymatic hydrolysis and the properties of peptides were affected by the solubility of proteins. SDS-PAGE of the enzymatic hydrolysates is shown in Figure 2C, which indicates that the hydrolysis degree of S3 and S6 is much higher than in the other samples.

### 2.2. Identification of the Peptides in Hydrolysates

A total of 277 peptides were identified from *M. edulis* by UPLC-Q-TOF-MS; 109, 14, 116, 67, 37, 144, 19, and 36 peptides were identified from S1 to S8, respectively. However, 47 peptides were derived from the hydrolysate of S0. The number of peptides from S1, S3, S4, and S6 was significantly higher than for S0. Over 70% of the peptides from S0 were also identified in all the other tissues separately. All these results indicated that more peptides would be identified if the samples were pretreated by separating the different tissues from *M. edulis* rather than that of the whole part. The number of peptides identified from S6 was the highest among all the samples, and the studies on the adductor muscle were also more extensive in recent years [18,19,20], which indicated that the adductor muscle from *M. edulis* could be a potential source of anticoagulant peptides.

### 2.3. Activity Prediction and Molecular Docking of Peptides

There were 25 peptides that showed higher scores (>50), listed in Table 1. The number of peptides derived from S0 to S8 was 5, 9, 6, 11, 7, 6, 12, 5, and 5, respectively. The results showed that the adductor muscle may contain more bioactive peptides.

**Figure 3:**
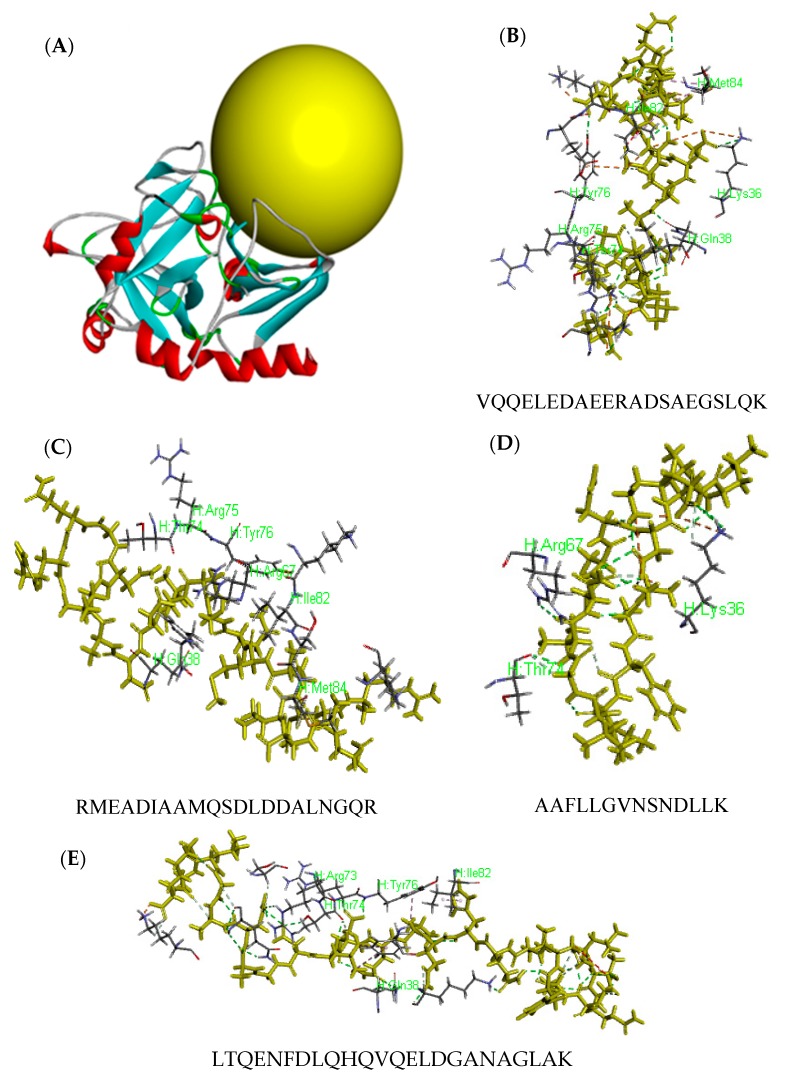
Molecular docking for the interactions of peptide against Thrombin. Molecular structure of thrombin from PDB database (**A**), the yellow sphere showed the docking position; VQQELEDAEERADSAEGSLQK (**B**), RMEADIAAMQSDLDDALNGQR (**C**), AAFLLGVNSNDLLK (**D**), and LTQENFDLQHQVQELDGANAGLAK (**E**). The peptides were marked with yellow sticks; the other sticks are the interactive amino acids of thrombin.

The CDCOCKER docking simulation was used to elucidate the molecular mechanisms of interactions between human thrombin and peptides from *M. edulis*. Every peptide has 10 molecular antagonists as docking poses for the docking simulation, as shown in Figure 3A. A peptide named TYS (containing hirudin fragment 54–62) was combined with thrombin and played an important role in inhibiting thrombin. The peptides derived from *M. edulis* proteins may show inhibitory activity against thrombin with a similar mechanism if the peptide and hirudin showed a similar interaction domain with thrombin (TYS). The yellow sphere was defined as the active site and binding site of peptides in Figure 3B. The score of TYS was 170.13. The number of peptides with a higher score (>170) derived from S0 to S8 was 7, 45, 33, 34, 16, 3, 55, 1, and 3 (Table 2), respectively. The percentage of peptides from the adductor muscle was much higher, so the antithrombotic activity of S6 was determined in the following study.

Three peptides, VQQELEDAEERADSAEGSLQK (P1), RMEADIAAMQSDLDDALNGQR (P2), and AAFLLGVNSNDLLK (P3), might be more active due to the higher evaluation levels of activity by both Peptide Ranker and molecular docking. The interaction of antithrombotic peptides with thrombin was shown in Figure 3C. Amino acids combined from the thrombin of P1, P2, and P3 were Lys36-Gln38-Thr74-Arg75-Tyr76-Ile82-Met84, Gln38-Arg67-Thr74-Arg75-Tyr76-Ile82-Met84, and Lys36-Arg67-Thr74, respectively. As is well known, Phe34-Leu65-Arg73-Thr74-Arg75-Tyr76-Glu80-Lys81-Ile82 is the active site 2 in the thrombin molecule [21], and Lys36-Arg73-Arg77-Lys149E in the thrombin molecule works as the binding motif to recognize fibrinogen [22]. The number of combined amino acids from P1–P3 was 5, 4, and 2; their scores were 303.38, 220.49, and 171.47. These results indicated that the more essential amino acids were combined, the higher the score and the stronger the activity [23]. The score of LTQENFDLQHQVQELDGANAGLAK was the highest. However, there were only 5 interactive amino acids involved in the active center of thrombin include, Gln38-Arg73-Thr74-Tyr76-Ile82, which was less than that of P1. Moreover, P1 was derived from the adductor muscle, P2 was derived from the foot and adductor muscle, and P3 was derived from the byssus, pedal retractor muscle, and adductor muscle. Therefore, the adductor muscle may be a potential source for producing peptides with anticoagulant activity.

### 2.4. Anticoagulant Activity Determination

**Figure 4:**
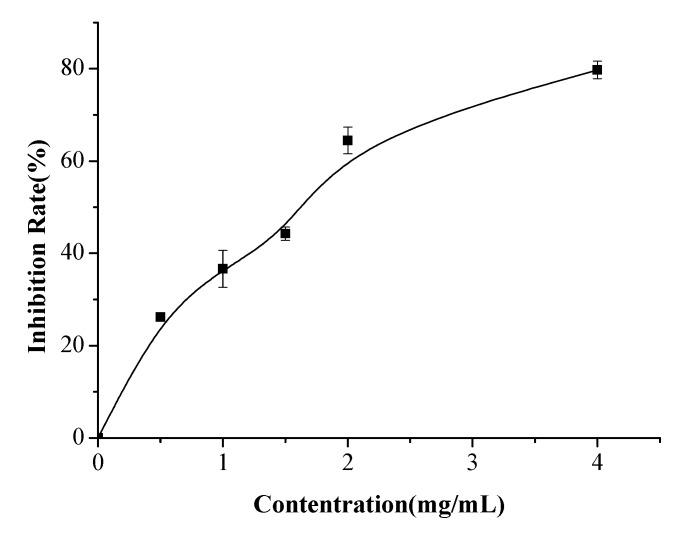
Anticoagulant activity IC50 of enzymatic hydrolysate from adductor muscle. Five different concentrations (0.5~4 mg/mL) were investigated, as measured by a microplate reader.

Anticoagulant activity IC_50_ values of hydrolysate from adductor muscle (0.5~4 mg/mL) were determined as shown in Figure 4. Results showed that IC_50_ was 1.49 mg/mL according to the fitted equation (y = 2.52 + 39.27x − 4.99x^2^). Moreover, the anticoagulant activity of the samples, foot, byssus, pedal retractor muscle, mantle, gill, and other parts at the same concentration were determined, and the inhibition rate was 43.14 ± 1.29(%), 48.50 ± 0.5(%), 27 ± 2.6(%), 22.55 ± 3.7(%), 19.71 ± 2.8(%), and 24.58 ± 0.81(%), respectively. These results indicated that the hydrolysate from the adductor muscle showed a higher anticoagulant activity than other tissues.

## 3. Materials and Methods

### 3.1. Materials and Chemicals

Bicinchonininc acid (BCA) and Cleanert S C18-N solid phase extraction (SPE) column were purchased from Beyotime (Beijing Baoxidi Science & Technology Co., Ltd., Beijing, China); Thrombin (EC 3.4.21.5), formic acid (FA), and acetonitrile (ACN) were purchased from Sigma-Aldrich Co. (St. Louis, MO, USA); Trypsin (EC3.4.21.4, 2.5 × 10^5^ U/g) was purchased from Solarbio (Beijing, China). All other chemicals used in this study were of analytical grade.

### 3.2. Split of Mytilus edulis Organisms

*M. edulis* was manually split into eight parts, i.e., the foot, byssus, pedal retractor muscle, mantle, gill, adductor muscle, viscera, and other parts; these were numbered S1, S2, S3, S4, S5, S6, S7, and S8, respectively, and the Whole *M. edulis* was named S0. The samples were freeze-dried with a vacuum freeze dryer (Ningbo Scientz Biotechnology Co. Ltd., Ningbo, China). The consequent powders were also treated with a ball-milled machine (RETSCH Verder Shanghai Instruments and Equipment Co., Ltd., Shanghai, China), and stored in a dry dish at 4 °C.

### 3.3. SDS-PAGE

The protein content of the samples was determined by the Kjeldahl method [24,25]. SDS-PAGE was carried out using AE-8135 (ATTO CORPORATION, Taito-ku, Japan). SDS-PAGE was performed using 10% polyacrylamide resolved gel (pH = 8.8) and 5% stacking gel (pH = 6.8). The protein bands were stained with Coomassie brilliant blue R-250. Premixed Protein Standard (44.3–200 kDa) of Takara Co. Ltd. (Dalian, China) was used for finding the relative molecular weight. The samples were mixed with 10 µL of electrophoretic loading buffer and heated for 5 min in a boiling water bath [26,27]. Different samples were separated by centrifugation at 8000 rpm for 15 min.

### 3.4. Enzymatic Hydrolysis by Trypsin

The enzymatic hydrolysis was conducted at 45 °C and pH 8.5. The pH was kept constant at 8.5 using 0.1 M NaOH as a regulator [1]. Once the optimum pH and temperature conditions were achieved, the enzyme trypsin (activity ≥ 5 U/mg) was added [28,29]. After 3 h, trypsin was heated–deactivated at 100 °C for 10 min in a water bath. The leaching effect of the protein from S0 to S8 is demonstrated by SDS-PAGE. The protein concentration was determined by BCA method [30].

### 3.5. Peptide Identification by UPLC-Q-TOF-MS/MS

The samples were processed with Cleanert S C18-N Solid phase extraction column [31]. Freeze-dried peptides were dissolved in 0.1% FA (soluble in water) and filtered through a 0.22-μM microporous filter membrane (Millipore, Billerica, MA, USA) before detection by mass spectrometry. Chromatographic separation was carried out at a flow rate of 0.4 mL/min with an injection volume of 15 μL on a C18 column (150 × 3 mm, 3 μm particle size). Peptides were separated using 0.1% FA in ultrapure water (solvent A) and 0.1% FA in ACN (solvent B) at a constant temperature of 25 °C. The gradient elution program was as follows: (i) 0 min 90% A; (ii) 0–2 min 75% A; (iii) 2–10 min 50% A; (iv) 10–20 min 40% A; (v) 20–35 min 60% A; (vi) 35–40 min 90% A [32,33].

Peptides were analyzed using an ESI-MS/MS (Bruker Co. Ltd., Bremen, Germany) with an ion source of ESI coupled with LC system (Thermo Fisher Scientific Co. Ltd., Waltham, MA, USA). The molecular mass and amino acid sequence of the peptides were determined by Mascot searching as follows: (i) The protein database was from the National Center for Biotechnology Information (https://www.ncbi.nlm.nih.gov/); (ii) the enzyme was set as trypsin; and (iii) the significance threshold was *p* < 0.05. The peptides were identified by database matching as well as the manual interpretation of its MS/MS spectrum, and the ion score of 35 was regarded as the identifying threshold [34].

### 3.6. Activity Prediction by Peptide Ranker

The activity of the peptides in samples 0–8 was predicted by the software of Peptide Ranker (http://bioware.ucd.ie/~compass/biowareweb/Serverpages/peptideranker.php). Peptide Ranker is a kind of database that provides certain classes of bioactive peptides with specific structural features that endow their particular functions by different classes of peptides. It concluded that there are general shared features of bioactive peptides across different functional classes, indicating that computational prediction may accelerate across many functional classes. The implementation of the predictive method was used to identify among a set of peptides [35]. The peptides with a score of more than 0.5 were considered to be positive results in the present study.

### 3.7. Molecular Docking

Molecular docking of the estimated anticoagulant peptides with thrombin were carried out using Discovery Studio 2017 software (Neotrident Technology Ltd., Beijing, China) according to the method described with some modifications [18]. The structure of peptide was processed and the energy minimized using the steepest descent and conjugate gradient techniques [36]. The corresponding receptor protein was downloaded from the PDB database (http://www.rcsb.org/pdb/home/home.do) and also treated by completing the missing amino acids, removing water molecules, and so on [37]. Docking was performed using the CDOCKER docking tool of Discovery Studio software. The best ranked docking pose of peptides in the active site of thrombin was obtained according to the score and binding-energy value [36].

### 3.8. Determination of Antithrombotic Peptides

A microplate reader was set to a wavelength of 405 nm at 37 °C. The fibrinogen, thrombin, and the samples were all dissolved in 0.05 M Tris-HCl (pH 7.2) containing 0.154 mM sodium chloride. Then, 140 μL of 0.1% fibrinogen solution and 40 μL of samples with different concentrations were added into the plate wells, mixed, and the absorbance of the sample blank was measured. Furthermore, 10 μL of thrombin (12 U/mL) were added and incubated at 37 °C. Finally, the absorbance was measured after 10 min [36,38,39,40]. The control group contained 40 μL of Tris-HCl buffer instead of the sample. The inhibition rate was calculated according to the following Equation (1), where *C*, *Cb*, *S*, and *Sb* represent the absorbance of control, control blank, sample, and sample blank, respectively.

(1) $$Inhibitory\;Rate\;(\%)=\frac{\left(C-Cb)-(S-Sb\right)}{C-Cb}\times 100\%$$

### 3.9. Statistical Analysis

Values were expressed as the mean ± SD (*n* ≥ 3). Following the assessment of significant differences between samples by one-way analysis of variance (ANOVA), the level of significance was set at *p* < 0.05. All statistical tests were conducted using SPSS software 19.0 (SPSS Inc., Chicago, IL, USA).

## 4. Conclusions

Different peptides components would be produced in the protein hydrolysates of the different tissues from *M. edulis*, which was digested by trypsin. Compared with the sample of blue mussels as a whole, many more peptides can be identified by UPLC-Q-TOF-MS if the tissues are identified separately. The IC_50_ of the hydrolysate from the adductor muscle of *M. edulis* was 1.49 mg/mL. The antithrombotic activity of different hydrolysates of *M. edulis* proteins can probably be attributed to the bioactive peptides in them, such as VQQELEDAEERADSAEGSLQK, RMEADIAAMQSDLDDALNGQR, and AAFLLGVNSNDLLK and so on; these peptides have a relative stronger affinity with thrombin (PDB: 2BVR). The present study may provide new ideas and technology on the screening or identification of peptides with anticoagulant activity on a large scale.

## Figures and Tables

**Table 1:** Peptides released from *Mytilus edulis* proteins hydrolyzed by trypsin.

Sequence	Length of Peptide	Molecular Weight	Tissues	Peptide Ranker
GPAGIIGLIGPK	12	857.4555	S1, S2, S3	0.83
GPIGPAGGKGPTGPK	15	1289.7138	S2	0.83
NDFDKDFFK	9	1174.5336	S6	0.78
AGLQFPVGR	9	569.3174	S0, S3, S4, S5, S6, S7, S8	0.76
GYSAELFR	8	792.3822	S1	0.71
SPNFTKPGK	9	801.4345	S1	0.7
GPTGPQGLR	9	881.4739	S1, S2	0.68
FPGQLNADLR	10	1129.5894	S3, S5	0.67
LAVNMVPFPR	10	1029.5439	S0, S3, S4, S5, S6, S7, S8	0.67
SYSAELFR	8	971.4715	S3, S4, S6	0.65
TATSPFFK	8	897.4602	S3, S4, S7, S8	0.65
KLAVNMVPFPR	11	1270.7253	S0	0.64
AVFPSIVGRPR	11	1197.7004	S0, S1, S2, S3, S4, S5, S6, S7, S8	0.62
LSHYAFSSLR	10	1180.4689	S5	0.62
AAFLLGVNSNDLLK	14	1473.8192	S3, S6	0.59
GIQGPEGELGPVGK	14	1337.7210	S2	0.58
TLYGFGG	7	713.3405	S5	0.58
WSYAPQSR	8	993.4698	S1, S3, S4, S6	0.58
VQQELEDAEERADSAEGSLQK	21	2331.0806	S6	0.57
AGFAGDDAPR	10	975.4435	S1, S2, S3, S4, S6, S7, S8	0.56
SFVNDIFER	9	824.5024	S0	0.56
NLNADVDSVRESLEEEQESKSDLQR	25	2889.364	S1	0.55
SPNFGRPGNASK	12	1230.6178	S3, S6	0.55
ALAADINLR	9	956.5409	S6	0.53
RMEADIAAMQSDLDDALNGQR	21	2319.064	S1, S6	0.5

Note: S0—Whole tissue; S1—Foot; S2—Byssus; S3—Pedal retractor muscle; S4—Mantle; S5—Gill; S6—Adductor muscle; S7—Viscera; S8—Other parts. Peptides with a score greater than 0.5 were considered to be positive results in the present study.

**Table 2:** Molecular docking of predicted antithrombotic peptides.

Peptide	Length of Peptide	Tissue(s)	-CDOCKERENERGY
LTQENFDLQHQVQELDGANAGLAK	24	S1	311.73
VQQELEDAEERADSAEGSLQK	21	S6	303.38
TLADLQKEEDKVNHLNK	17	S1	282.78
MEKENALDRAEQLEQK	16	S1	272.87
KKLEQDINELEMALDTSNR	19	S1	270.74
SIQTENDLDNTQTQLQDVQAK	21	S6	264.94
AKLESTLDEMEDNLER	16	S1	255.701
ITIQQELEDARSLLEHAER	19	S1, S2, S3, S6	254.26
LADELRQEQDNYKNAESLR	19	S6	251.11
KLEQDINELEMALDTSNR	18	S1	249.98
HQEALNDLTDQLEHMGK	17	S1, S2, S3, S4, S6	241.79
RRHQEALNDLTDQLEHMGK	20	S1, S6	238.67
NRLQGELDDLLIEVER	16	S1	237.02
VKELQTEIDTAHTEAR	16	S1	236.54
DLEETTLQHEAQVSSLR	17	S6	236.102
MIEEAEDVASITMNKYR	17	S1, S6	235.72
RHQEALNDLTDQLEHMGK	18	S1, S2, S3, S4 S6	232.22
WIAEEADKKYEEAAR	15	S1, S2, S3, S6	231.80
AAVLEYLAAEVLELAGNAAR	20	S0	231.28
LLDEEDAASELEGLKK	16	S1	230.16
NQLIIEIDSLQAMNDGLQK	19	S2, S3, S6	228.63
LADELRQEQDNYK	13	S2, S3, S6	227.66
QNLQVQLAAIQSDYDNLNAR	20	S6	227.36
VIDLEEQLTVVGANIK	16	S1, S6	226.86
NLAEEIHELTEQLSEGGR	18	S6	226.02
AAEERADRLQAEVNR	15	S2, S3, S6	225.98
IRELEDSLDSEREMR	15	S1, S4	225.89
IRDLENELEADQRR	14	S2, S3, S4, S6	225.40
MIEEAEDVASITMNK	15	S6	223.61
ELEDSLDSEREMR	13	S2, S3, S6	222.18
RMEADIAAMQSDLDDALNGQR	21	S1, S6	220.49
ENALDRAEQLEQK	13	S1, S6	220.44
EVDRLEDELLTEK	13	S6	219.35
EITVRLEEAEAFAQR	15	S6	217.95
TFDREGQGYISGAEMR	16	S1	217.67
KLAITEVDLERAEAR	15	S1	214.16
ATQEVVEELEGVKR	14	S1, S2, S3	213.487
LTEVQLQVTALTNDKR	16	S2, S3, S6	213.42
LQGELDDLLIEVER	14	S1	213.06
AVFVDLEPTVVDEVR	15	S5	212.988
VQFNLKDYQSSANVKHAVDK	20	S4	212.98
IRDLENELEADQR	13	S1, S2, S3, S4, S6	212.26
HQGVMVGMGQKDSYVGDEAQSKR	23	S1	212.01
ELQTEIDTAHTEAR	14	S1	211.61
LEDAMGTSTTVSEVSR	16	S6	211.59
TLQGEMAQQDEQISK	15	S1	211.51
IAIIITDGKPTDINATQR	18	S2, S3, S4	211.34
SGVLVRPK	8	S0, S8	210.81
MSADSKIDALEGSNSR	16	S1, S2, S3, S6	209.52
DLENELEADQRR	12	S2, S3, S6	209.289
AQYEETSDTIEALRR	15	S1	209.038
DLYANTVLSGGTTMFPGIADR	21	S6	207.485
QLDDTRNQLSVSER	14	S6	206.48
LTGELEDLGIDVER	14	S6	206.25
QIAEHEQEIQSLTR	14	S6	205.14
ELDDVQSQLSHSMK	14	S1	204.91
QLEDAEHTIGSLTK	14	S1	204.72
ELEGELDSEQRR	12	S6	203.33
LAEAEQAAEAANAK	14	S1	202.27
IRELEDSLDSER	12	S2, S3, S4	202.17
ALDSMQASLEAEAK	14	S2, S3, S6	202.096
QVAELTSITDQLTMK	15	S6	201.63
INELAAQVSSAQAQKR	16	S1	201.27
DKSALTSQLEEAKR	14	S1	201.151
KNAENELGEVTVR	13	S0, S2, S3, S4, S6	200.88
LLSGVTIAQGGVLPNIQAVLLPK	23	S0	200.55
AKIEDDYNSLQK	12	S1, S6	198.5
SYYDTSREENDIRR	14	S6	197.19
SYELPDGQVITIGNER	16	S0,S1, S2, S3, S4, S6, S7, S8	196.76
VTDLQSELENAQK	13	S2, S3, S6	196.65
DLENELEADQR	11	S6	196.13
LDLAGRDLTDYLMK	14	S1, S2, S3, S4, S6	191.19
KVGINYQPPTVVPGGDLAK	19	S0	190.22
DIEDLETTLAK	11	S1	189.759
SALYEDTFIPEVIRPR	16	S2, S3, S6	188.36
LEDDQSLIAQLQR	13	S6	188.31
YEEESENASSLR	12	S2, S3, S4, S6	188.24
MSATFIGNSTAIQELFKR	18	S5	184.40
NAENELGEVTVR	12	S6	183.23
AMSIMNSFVNDIFER	15	S0, S2, S3, S4, S5, S8	178.809
DSYVGDEAQSKR	12	S1, S2, S3, S4	177.848
SALTSQLEEAKR	12	S1	177.63
DSYVGDEAQSK	11	S1, S2, S3	177.572
KRITIQQELEDAR	13	S2, S3, S6	177.35
KAQSLIDEAEQR	12	S2, S3, S4	177.22
AQSLIDEAEQR	11	S4	176.453
ETVQASDEDRR	11	S6	176.32
QLENENAALQK	11	S2, S3	175.83
KMEGENSEMKR	11	S1	174.67
QEYDESGPSIVHR	13	S6	174.00
LTDEQVDDIIR	11	S6	173.52
ATQEAVEDLER	11	S2, S3	173.43
KLAITEVDLER	11	S6	173.15
SKLQSEVTEINR	12	S1	172.71
ELEDSLDSER	12	S3, S6	172.60
ITIQQELEDAR	11	S2, S3, S6	171.94
LTDMIDKLQSK	11	S1	171.79
AAFLLGVNSNDLLK	14	S2, S3, S6	171.476
SLENTIAELQHK	12	S2, S3, S6	170.94
ENKNLADEIR	12	S1	170.32

Note: S0—Whole tissue; S1—Foot; S2—Byssus; S3—Pedal retractor muscle; S4—Mantle; S5—Gill; S6—Adductor muscle; S7—Viscera; S8—Other parts. Peptides with scores greater than 170 were considered to be anticoagulant peptides in the present study.
